# Associations of multiple toxic metal exposures with metabolic dysfunction-associated fatty liver disease: NHANES 2011–2018

**DOI:** 10.3389/fnut.2023.1301319

**Published:** 2023-12-04

**Authors:** Yuguang Li, Zefeng Liu, Yu Chang, Naifei Chen, Rong Zhang, Xiangliang Liu, Wei Song, Jin Lu

**Affiliations:** ^1^Cancer Center, The First Hospital of Jilin University, Changchun, China; ^2^Department of Hepatobiliary Pancreatic Surgery, The Second Hospital of Jilin University, Changchun, China; ^3^Department of Gastroenterology, The First Hospital of Jilin University, Changchun, China

**Keywords:** MASLD, mercury, manganese, lead, selenium, cadmium, NHANES

## Abstract

**Background:**

The occurrence of metabolic dysfunction-associated fatty liver disease (MASLD) is driven by multiple factors including obesity, hypertension, dyslipidemia, and insulin resistance. However, epidemiological research investigating the association between metal exposure and MASLD occurrence remains limited.

**Methods:**

We conducted a large cross-sectional study with 6,520 participants who were involved in the National Health and Nutrition Examination Survey (NHANES) between 2011 and 2018. Using generalized linear regression, we examined the relationship between five heavy metals (mercury, manganese, lead, selenium, cadmium) and MASLD. Furthermore, restricted cubic spline models and weighted quantile sum (WQS) analysis were employed to characterize the exposure-response relationship between the five metals and MASLD.

**Results:**

Higher blood selenium levels were associated with an increased likelihood of MASLD among US adults. Blood lead exposure was also positively correlated with MASLD risk. However, there was no significant association observed between blood cadmium, mercury, manganese levels, and MASLD risk. Among the five metals, blood cadmium exposure accounted for the highest proportion of MASLD risk.

**Conclusion:**

Our study indicated the significant association between blood cadmium and lead exposure levels and the occurrence of MASLD in a representative sample of US adults.

## 1 Introduction

Nonalcoholic fatty liver disease (NAFLD) is characterized by hepatic steatosis without excessive alcohol use, and has become a major public health issue with the pandemic of obesity and type 2 diabetes worldwide (1). NAFLD represents a spectrum of progressive liver conditions, which can progress from simple steatosis to nonalcoholic steatohepatitis, advanced fibrosis, cirrhosis, and even hepatocellular carcinoma (2). The global prevalence of NAFLD is estimated to be 25%, and up to 45% in developed countries (3). In June 2023, NAFLD was officially renamed as metabolic dysfunction-associated fatty liver disease (MASLD) with modified diagnostic criteria (4). NAFLD, a common disease with a prevalence of up to 40% in the general population, lacks sufficient public awareness regarding its diagnosis and related symptoms. Renaming it as MASLD can better reflect the pathophysiological processes of the disease and improve public understanding of this condition. The occurrence of MASLD is driven by various metabolic risk factors such as obesity, insulin resistance, hypertension, hyperlipidemia and hyperglycemia (1). Meanwhile, accumulating evidence suggests that chronic exposure to environmental pollutants may also contribute to MASLD pathogenesis, in addition to genetic and metabolic factors (5, 6).

Humans are exposed to various metals including essential and toxic elements from numerous sources such as air, food, water, and consumer products (7). Essential metals like zinc, copper and selenium are required for biological functions and act as integral components of proteins and enzymes in human body. However, excessive levels of essential metals can also elicit toxicity (8). Nonessential metals such as arsenic, cadmium, lead and mercury have no known physiological roles and their accumulation in the body can be hazardous to human health (9). After absorption, metals circulate in the blood and distribute to different tissues including the liver, which is the primary site of xenobiotic biotransformation and metabolic homeostasis (5). Various metals have been shown to cause hepatic toxicity through mechanisms such as inducing oxidative stress, disrupting cellular signaling, altering lipid metabolism and stimulating inflammation (10–12). Recent studies also provide evidence implicating toxic metals exposure in the development of hepatic steatosis and nonalcoholic fatty liver injury in animals (13–15). However, epidemiological studies that link metals exposure to MASLD risk are still limited.

In this study, we analyzed the data from the continuous National Health and Nutrition Examination Survey (NHANES) during 2011–2018 to evaluate the associations of blood levels of manganese, lead, cadmium, mercury and selenium with MASLD prevalence in American adults. Advanced statistical approaches including restricted cubic spline and weighted quantile sum models were employed to characterize the complex exposure-response relationships. Elucidating the role of metals in MASLD development will help identify the modifiable environmental risk factors and provide prevention strategies.

## 2 Methods

### 2.1 Study population

**Figure 1:**
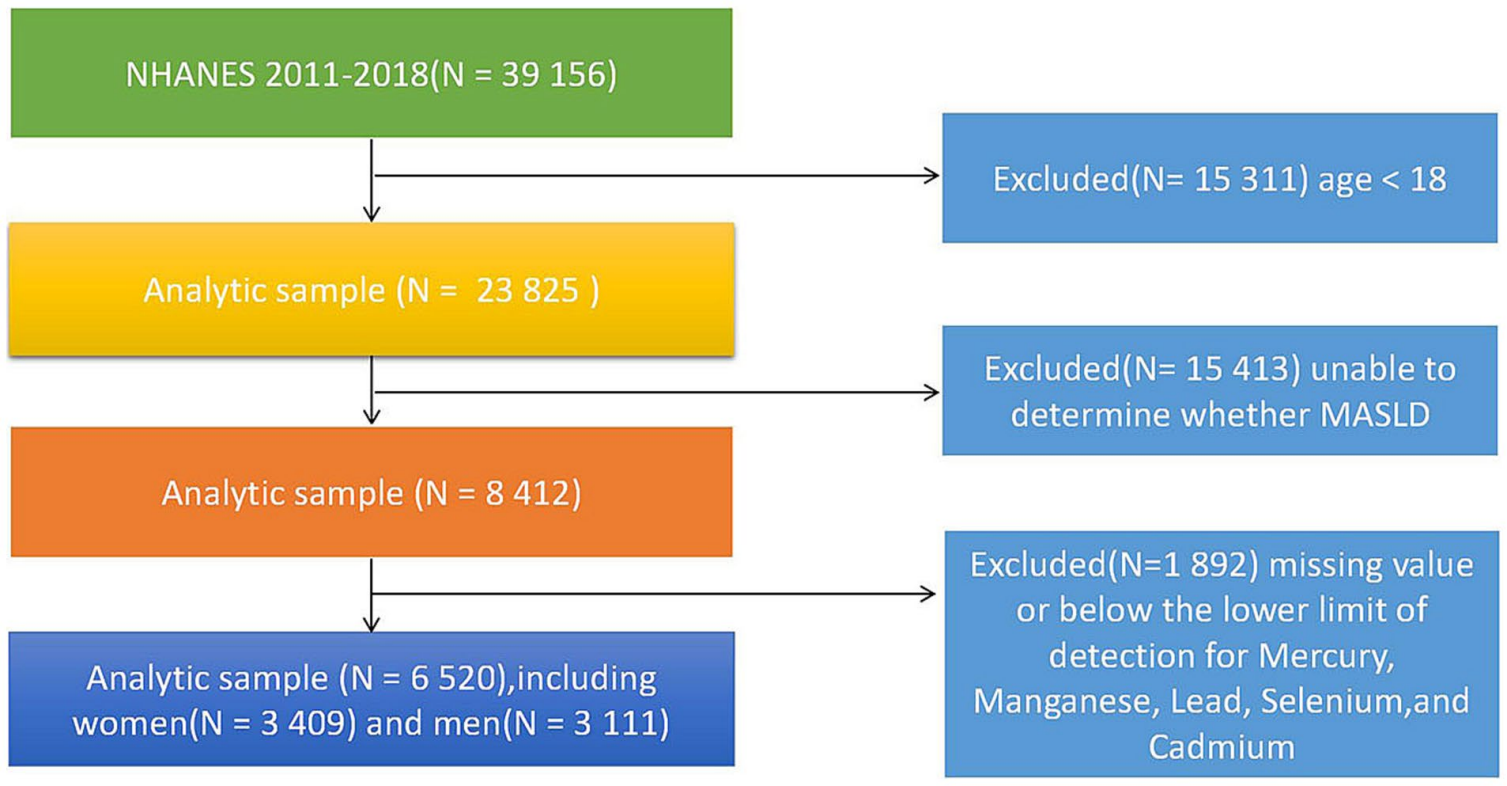
The flow diagram of the study participants, from NHANE 2011–2018.

As a cross-sectional study, the data were obtained from NHANES (The National Health and Nutrition Examination Survey). NHANES is a major project of the National Center for Health Statistics (NCHS), overseen by the Centers for Disease Control and Prevention (CDC), which provides relevant participant data. This large-scale cross sectional survey is designed to provide cross-sectional data on the health and nutrition of the adults and children in the United States, which can be used for research, policy development, and public health planning. The study has obtained approval from the National Center for Health Statistics Ethics Review Board, and written informed consent was obtained from each participant. A total of 39,156 participants from NHANES 2011–2018 were included in the study. Participants under the age of 18 (*n* = 15,331), for which data on blood lead levels were not provided, were excluded, as well as those who do not meet the diagnostic criteria for MASLD (*n* = 15,413), and those for whom blood trace elements (mercury, manganese, lead, selenium, cadmium) could not be obtained or detected (*n* = 1,892). Ultimately, a total of 6,520 participants were included in the final analysis, consisting of 3,409 males and 3,111 females. Please refer to Figure 1 for specific details.

### 2.2 Assessment of MASLD

The diagnostic criteria for metabolic-associated fatty liver disease (MAFLD) related to metabolic dysfunction are based on hepatic steatosis (accumulation of fat in the liver), combined with one of the following three criteria: overweight/obesity, type 2 diabetes mellitus, evidence of metabolic dysregulation. Metabolic dysregulation should include at least two of the following abnormalities: (i) Waist circumference ≥ 102/88 cm for men/women in Caucasians, or ≥ 90/80 cm for men/women in Asians; (ii) Blood pressure ≥ 130/85 mmHg, requiring specific medication; (iii) Plasma triglycerides ≥1.70 mmoL/L (≥150 mg/dL), requiring specific medication; (iv) Plasma high-density lipoprotein cholesterol <1.0 mmoL/L (<40 mg/dL) for men, or < 50 mg/dL (<1.3 mmoL/L) for women, requiring specific medication; (v) Fasting plasma glucose level of 5.6–6.9 mmoL/L or 2-h post-load glucose level of 7.8–11.1 mmoL/L, or glycated hemoglobin level of 39–47 mmol/mol; (vi) Steady-state assessment of insulin resistance score ≥ 2.5; (vii) High-sensitivity C-reactive protein level > 2 mg/L. Following these criteria, participants in the study were screened and divided into the MAFLD group and Non-MAFLD group. This information can be used in high-quality scientific publications (4).

### 2.3 Assessment of toxic metals

The whole blood specimens were collected and stored frozen at −30°C, and transported to the National Center for Environmental Health, Centers for Disease Control and Prevention, located in Atlanta, Georgia. The concentrations of trace elements in the blood were measured using an inductively coupled plasma (ICP) ionization source mass spectrometer (16, 17). The lower limit of detection (LLOD) for blood lead, cadmium, manganese, mercury, and selenium were 0.07 μg/dL, 0.10 μg/L, 0.99, 0.28, and 24.48 μg/L, respectively. The proportions of blood lead, cadmium, manganese, mercury, and selenium concentrations above the respective LODD were 82.23, 73.11, 100, 66.08, and 100%. For results below the LODD, the detection value (LODD/sqrt[2]) was used as a substitute. More specific details can be obtained through the website: https://wwwn.cdc.gov/Nchs/Nhanes/2017-2018/PBCD_J.htm#LBDBMNLC.

### 2.4 Statistical analysis

We categorized the levels of five trace elements in blood, namely lead, cadmium, manganese, mercury, and selenium, based on quartiles (Quartile 1: <25th percentile, Quartile 2: 25th–50th percentile, Quartile 3: 50th–75th percentile, Quartile 4: >75th percentile). Independent samples *t*-test was used to analyze the differences in trace element exposure between the Non-MASLD (Non-Metabolic Associated Fatty Liver Disease) group and the MASLD group as continuous variables, represented by mean ± variance. Chi-square test was used to assess demographic characteristics including gender (male, female), age (<65 years, ≥65 years), race (Mexican American, Non-Hispanic Black, Non-Hispanic White, Other Hispanic, Other race-including multi-Racial), poverty status (<1, 1–3, ≥3), educational status (low high school, high school, college or above), BMI (<25, ≥25), represented by N(%). Generalized linear regression models were employed to evaluate the relationship between blood trace elements and MASLD, yielding corresponding risk ratios, 95% confidence intervals, and depicted in forest plots. The model has been adjusted for multiple potential confounders, encompassing gender, age, race, educational level, poverty index, and BMI. Restricted cubic spline (RCS) plots were used to assess the non-linear and dose–response relationships between trace elements and MASLD. Weighted quantile sum (WQS) models were applied to evaluate the relationship between combined trace elements and MASLD and calculate their respective weights. The WQS model is considered as a statistical model that uses multiple regressions on high-dimensional datasets. By constructing weighted indices, it creates a composite outcome variable from multiple exposure variables. The model then assigns weights to each exposure variable, allowing for the evaluation of the relative importance of individual variables on the outcome. All statistical analyses were conducted using SPSS version 26.0 (IBM) or R Project for Statistical Computing (version 4.2.2). All tests were two-tailed, and *p*-values <0.05 were considered statistically significant.

## 3 Results

### 3.1 Baseline features

The flowchart of the participant screening process is shown in Figure 1. A total of 6,520 individuals were included in the study, with 4,310 in the Non-MASLD group and 2,210 in the MASLD group. The baseline characteristics of the participants are presented in Table 1. In the Non-MASLD group, there was a higher proportion of female participants, accounting for approximately 55.17%, while males accounted for 46.65%. In the MASLD group, there were more male participants, comprising around 53.35%, while females accounted for 44.83%. Both groups had a higher proportion of participants below the age of 65, with 79.26% in the Non-MASLD group and 70.59% in the MASLD group, whereas participants aged 65 or above accounted for 20.74 and 29.41%, respectively. Regarding the racial distribution in the MASLD group, 13.94% were Non-Hispanic Black, 38.96% were Non-Hispanic White, 20.63% were Mexican American, 12.17% were Other Hispanic, and 14.30% were of Other race-including multi-Racial. A larger proportion of participants in both groups had a poverty-income ratio between 1 and 3, with percentages of 42.26% in the Non-MASLD group and 45.01% in the MASLD group. The MASLD group had a higher proportion of participants with education below high school (26.92%). In terms of BMI, the MASLD group had a higher BMI, with 68.56% classified as obese (BMI > 30), while only 20.87% of the Non-MASLD group fell into this category. All these baseline characteristics showed significant differences between the two groups. Furthermore, in relation to the five trace elements measured as continuous variables, the MASLD group had higher concentrations of selenium and manganese in the blood compared to the Non-MASLD group, while mercury, lead, and cadmium showed the opposite pattern. Additional baseline characteristics can be found in Table 1.

**Table 1:** Characteristics of the 6,520 participants with and without MASLD.

Variable	Level	No MASLD	MASLD	*P*-value
n		4,310	2,210	
Sex (%)				
	Female	2,378 (55.17)	1,031 (46.65)	<0.0001
	Male	1,932 (44.83)	1,179 (53.35)	
Age (%)				
	[18, 65)	3,416 (79.26)	1,560 (70.59)	<0.0001
	≥65	894 (20.74)	650 (29.41)	
Race/ethnicity (%)				
	On-Hispanic Black	1,133 (26.29)	308 (13.94)	<0.0001
	On-Hispanic White	1,520 (35.27)	861 (38.96)	
	Mexican American	401 (9.30)	456 (20.63)	
	Other Hispanic	414 (9.61)	269 (12.17)	
	Other race—including multi-racial	842 (19.54)	316 (14.30)	
Poverty (%)				
	<1	851 (21.93)	460 (23.21)	0.0089
	[1, 3)	1,640 (42.26)	892 (45.01)	
	≥3	1,390 (35.82)	630 (31.79)	
Education (%)				
	Low high school	889 (20.64)	595 (26.92)	<0.0001
	High school	1,062 (24.65)	525 (23.76)	
	College or above	2,357 (54.71)	1,090 (49.32)	
BMI (%)				
	<25	1,877 (43.62)	119 (5.40)	<0.0001
	[25, 30)	1,528 (35.51)	574 (26.04)	
	≥30	898 (20.87)	1,511 (68.56)	
Selenium [mean (SD)]		192.518 (27.545)	195.778 (26.387)	<0.0001
Manganese [mean (SD)]		10.052 (3.758)	10.284 (3.880)	0.0195
Mercury [mean (SD)]		1.676 (2.783)	1.390 (2.645)	0.0001
Lead [mean (SD)]		1.420 (1.623)	1.411 (1.868)	0.8501
Cadmium [mean (SD)]		0.561 (0.614)	0.518 (0.613)	0.0069

MASLD, metabolic dysfunction-associated fatty liver disease. Mean ± SE for continuous variables: *P*-value was calculated by the weighted *t-*test. % (SE) for categorical variables: *P*-value was calculated by the weighted chi-square test.

### 3.2 The association between five trace elements and MASLD

**Figure 2:**
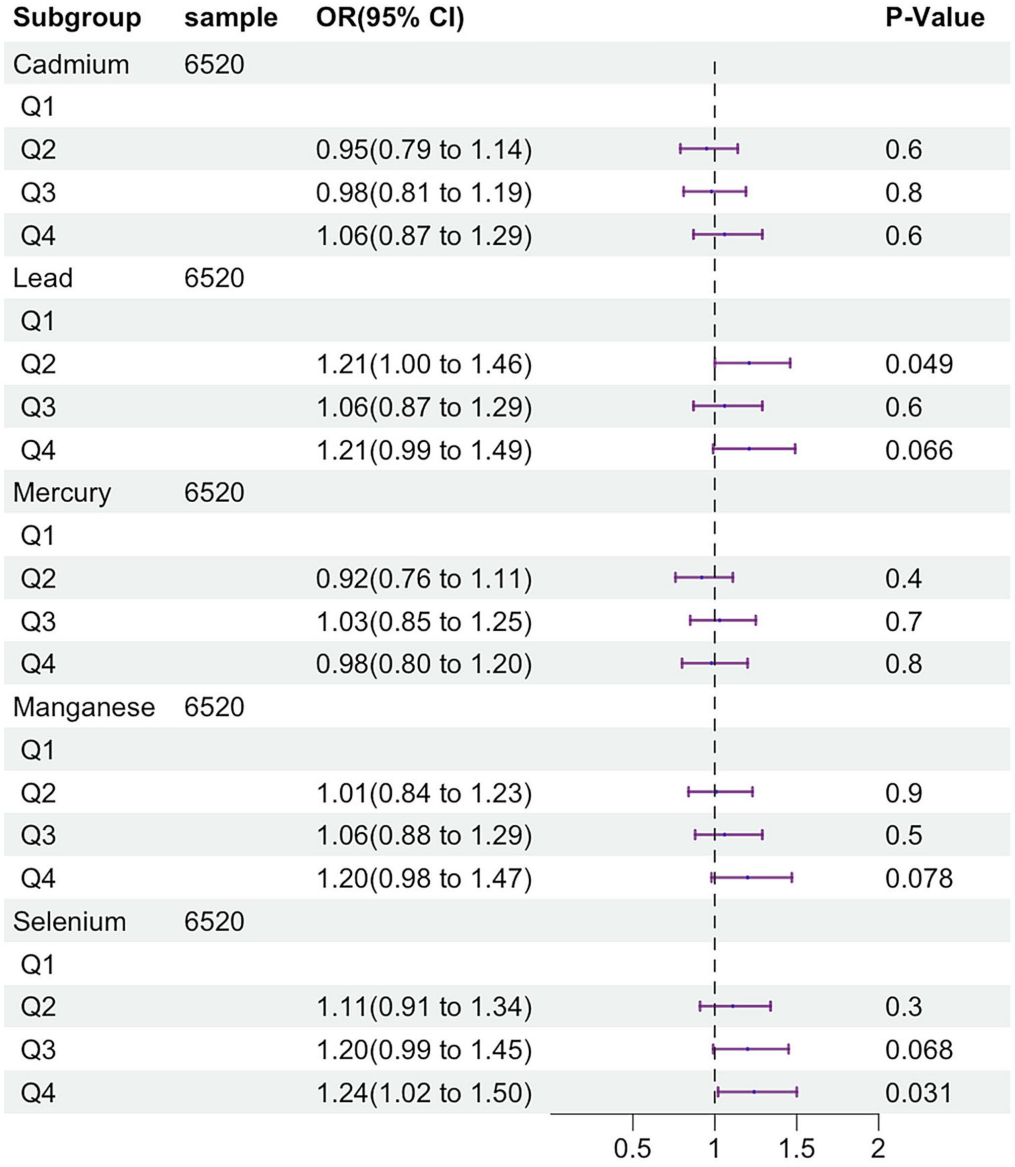
Association between blood trace mineral concentration (μmol/L) and MASLD risk, and adjusted for sex, age, race/ethnicity, education attainment, poverty income ratio and BMI.

By generalized linear regression, we evaluated the relationship between five trace elements and MASLD (Figure 2). In the adjusted linear model, compared to the Q1 group, selenium demonstrated a significant difference in both the Q3 and Q4 groups, indicating that higher selenium levels were associated with an increased risk of MASLD (Q3: OR 1.20, 95%CI 0.99–1.45, *p* = 0.068; Q4: OR 1.24, 95%CI 1.02–1.50, *p* = 0.031). Lead also showed a significant difference in the Q2 group compared to the Q1 group (Q2: OR 1.21, 95%CI 1.00–1.46, *p* = 0.049), although no positive results were observed in the Q3 and Q4 groups, the hazard ratios (HR) were greater than 1 (Q3: OR 1.06, 95%CI 0.87–1.29, *p* = 0.60; Q4: OR 1.21, 95%CI 0.99–1.49, *p* = 0.066), indicating a trend of higher lead levels being associated with an increased risk of MASLD. However, no significant differences were found for cadmium and mercury manganese.

### 3.3 Non-linear regression analyses

**Figure 3:**
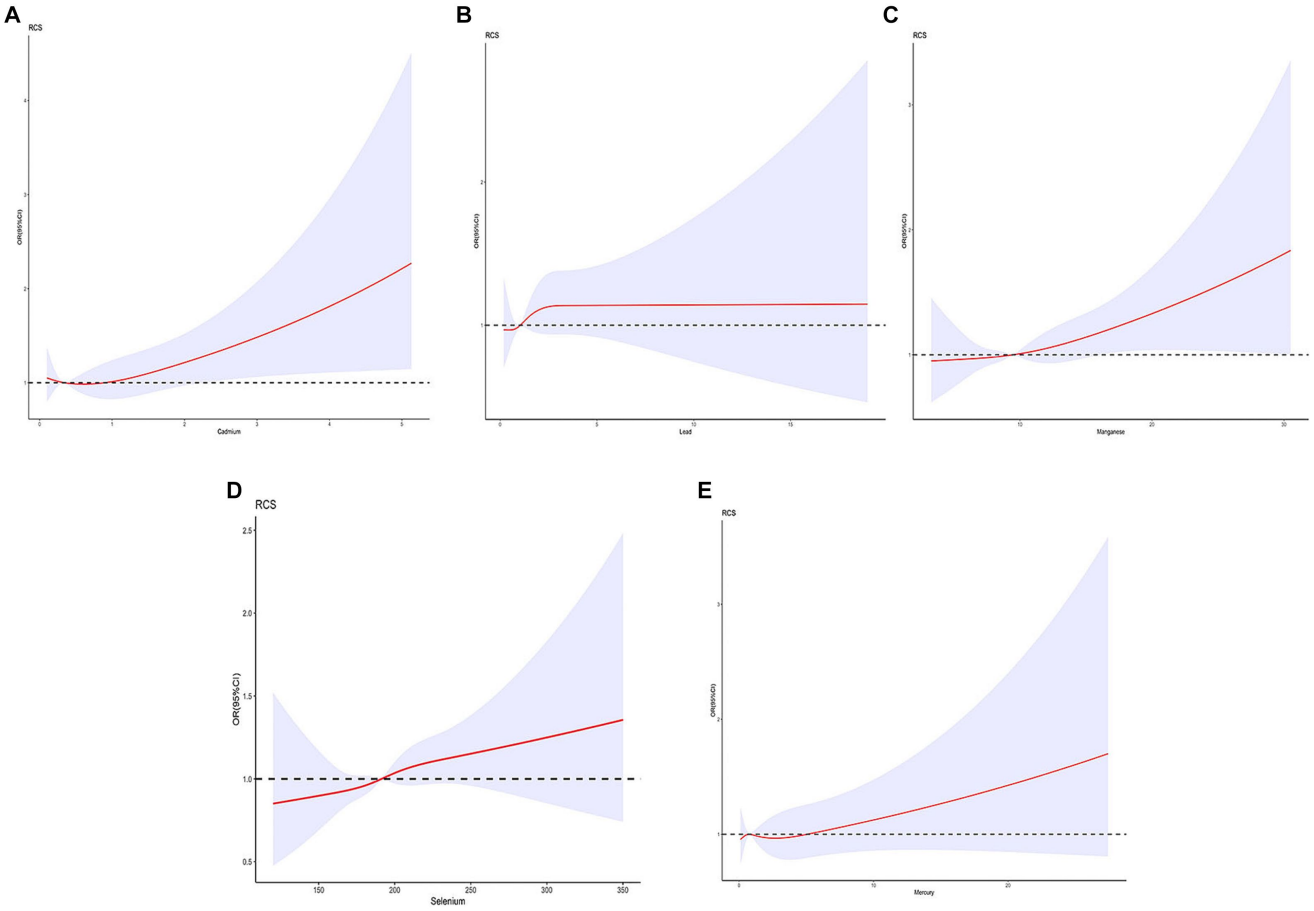
The non-linear relationship between blood cadmium (μg/L) **(A)**, lead (μg/dL) **(B)**, manganese (μg/L) **(C)**, selenium (μg/L) **(D)**, mercury (μg/L) **(E)** and MASLD risk, sex, age, race/ethnicity, education attainment, poverty income ratio and BMI were adjusted.

We also constructed restricted cubic splines (RCS) plots based on the aforementioned adjusted linear regression model to evaluate the relationship between five trace elements and MASLD (Figure 3). It was observed that the associations between the five trace elements and periodontitis were inconclusive, as all *p*-values were greater than 0.05. However, there was a positive trend between blood concentrations of manganese and selenium with the occurrence of MASLD. Specifically, for manganese, the overall *p*-value was 0.1172 and the non-linear *p*-value was 0.8022. As for selenium, the overall *p*-value was 0.2211, and the non-linear *p*-value was 0.8912.

### 3.4 Weights computation—WQS model

**Figure 4:**
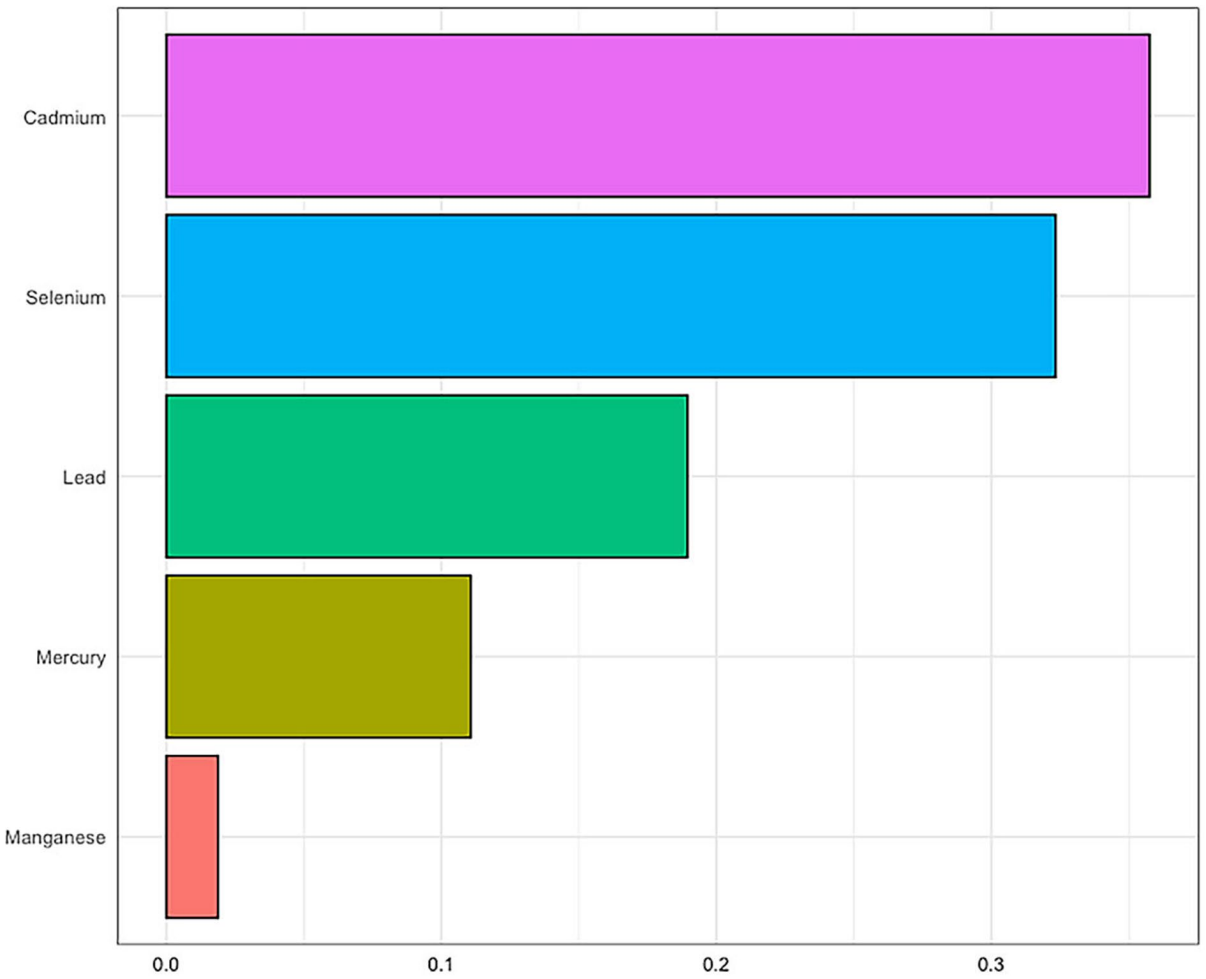
Weighted quantile sum analysis suggested potential synergistic interactions between blood trace mineral concentration and MAFLD risk.

**Figure 5:**
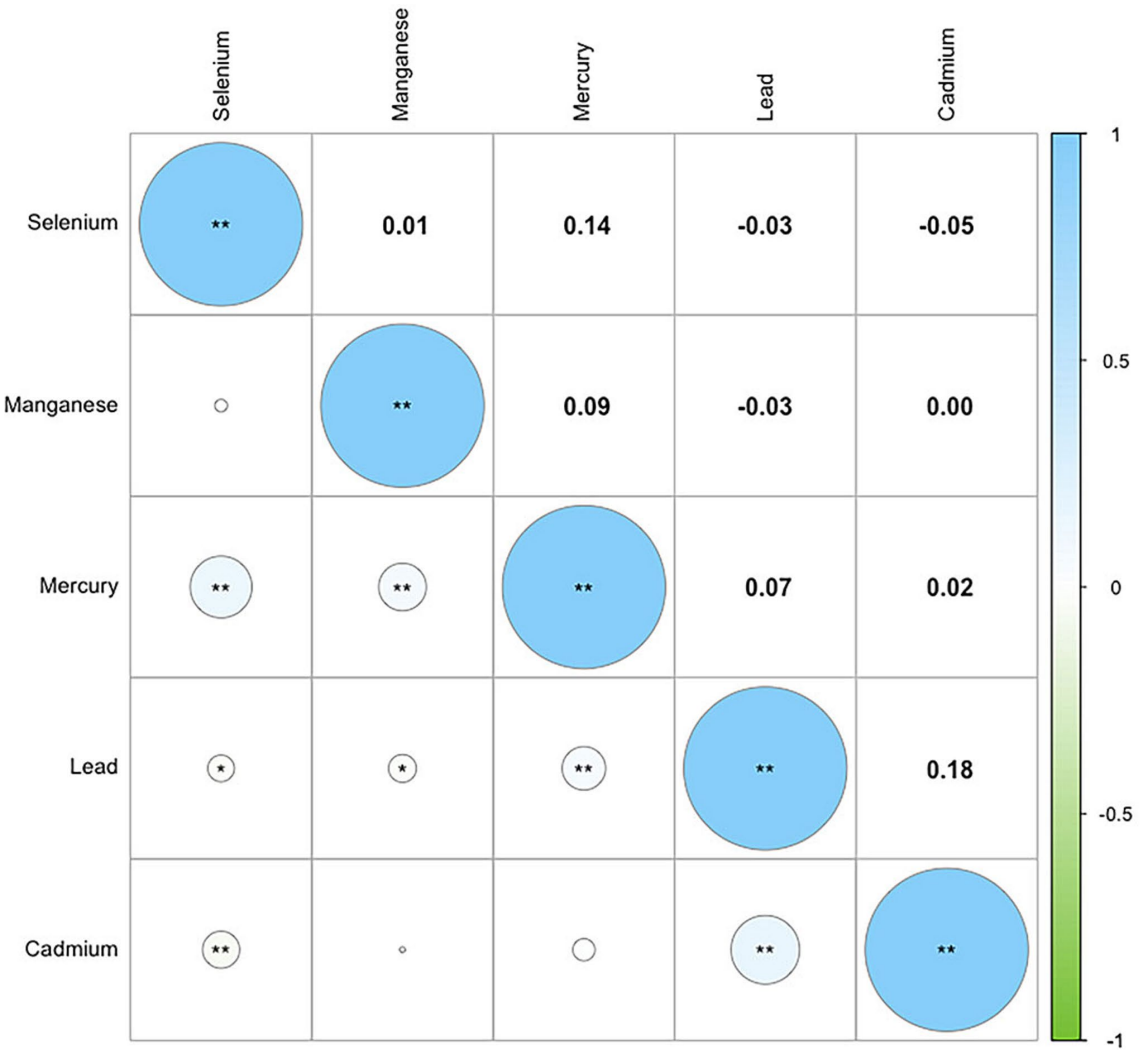
Pearson correlation coefficients between the exposures to the five metallic elements. * *p* < 0.05; ** *p* < 0.01.

To further explore the complex exposure-response relationship between metal elements and MASLD, we applied the Weighted Quantile Sum (WQS) model to evaluate the combined effects of multiple metals on MASLD. The analysis revealed a WQS index of 1.13 (95% CI: 1.00–1.28, *p* = 0.0454), indicating a significant synergistic interaction among different metals. Subsequent analysis of the weight proportions for the five trace elements showed that cadmium accounted for 35.76%, selenium accounted for 32.34%, lead accounted for 18.95%, mercury accounted for 11.07%, and manganese accounted for 1.88%. This suggests that exposure to cadmium may pose the greatest risk for MASLD. Further details can be found in Figure 4. The Pearson correlation coefficients between the exposures to the five metallic elements can be found in Figure 5.

## 4 Discussion

Metabolic dysfunction-associated steatotic liver disease (MASLD) is closely associated with obesity, diabetes and other metabolic disorders (4). MASLD may progress to steatohepatitis, fibrosis, cirrhosis, and even hepatocellular carcinoma (1). Environmental exposure to toxic metals is considered an important risk factor for MASLD in addition to metabolic factors (5, 6). These metals can participate in MASLD pathogenesis by disrupting glucose/lipid metabolism, inducing oxidative stress and inflammatory responses, and causing liver injury (10–12). In this nationally representative study, elevated blood selenium and lead levels were associated with increased MASLD risk in American adults. This provides novel epidemiological evidence linking metals exposure to MASLD.

In this nationally representative sample, we found that elevated exposure to selenium was associated with higher likelihood of MASLD in US adults. Lead exposure also showed a positive correlation with MASLD risk. Our results were in line with several previous animal studies. Selenium oversupplementation has been shown to induce hepatic lipid accumulation in mice (10). High dietary intake of lead could cause hepatic oxidative damage and inflammation in rats (12). However, epidemiological studies on their associations with NAFLD in humans are still very limited with inconsistent results (13, 15).

RCS and WQS models are advanced statistical approaches to characterize complex exposure-response relationships and cumulative effects from metal mixtures (12, 14). Application of these novel methods allowed comprehensive evaluation of multiple metals and their joint impact on MASLD risk. Our WQS analysis suggested potential synergistic interactions between different metals. Cadmium appeared to confer the greatest risk despite no significant independent association, suggesting metabolic interplays among metals. The advantages of RCS and WQS models should be considered in future environmental health studies.

While selenium is an essential trace nutrient, excessive selenium can elicit oxidative stress and lipid peroxidation, leading to liver damage (12). High selenium may upregulate selenoproteins and glutathione peroxidases, resulting in overproduction of hydrogen peroxide and oxidative injury (13). Selenium can also induce inflammatory cytokines by activating NF-κB and MAPK pathways (18). Lead is a ubiquitous toxicant capable of accumulating in the body and eliciting multi-organ toxicity. Potential mechanisms by which lead promotes MASLD include inducing hepatic insulin resistance, disrupting lipid metabolism, and causing oxidative stress (12). Lead exposure is associated with dysfunction of liver X receptors that regulate cholesterol homeostasis (15). Lead can also bind to antioxidants like glutathione and replace zinc in metalloenzymes, leading to oxidative damage (7). Additionally, lead can provoke inflammation by activating HIF-1α and TLR4 pathways (12, 14).

Although no independent association was found between cadmium and MASLD, our analysis suggested cadmium exposure significantly contributed to the metal mixture-associated MASLD risk. Cadmium accumulates in the liver and causes hepatotoxicity (19), potentially by impairing mitochondrial function, inhibiting antioxidant enzymes, inhibiting DNA repair and inducing cell apoptosis (17). Complex interactions likely exist between different metals that warrant further elucidation.

This population-based study provides novel epidemiological evidence linking environmental exposures to toxic metals with the risk of MAFLD in US adults. Advanced statistical approaches were used to comprehensively examine the relationships between multiple metals and their joint effects on MAFLD. The findings can aid in identifying environmental determinants, informing prevention strategies, and emphasizing the importance of reducing metal exposures to combat the emerging MAFLD epidemic worldwide. Moreover, this study offers insights into the role of environmental factors in the pathogenesis of MAFLD, which require further investigation.|.

However, our study still has significant limitations. Despite adjusting for multiple variables in the model, there may still be unmeasured confounding factors. These confounding factors were not considered or were deleted due to incomplete information in the NHANES database during model development. Furthermore, this study is cross-sectional and limited by its experimental design, as it did not explore the causal relationship between metal exposure and MAFLD further. Lastly, the data for this study were obtained from the NHANES database, and the participants were all US residents. Caution should be exercised when generalizing the research findings to other populations in different countries.

## 5 Conclusion

In summary, this study provides novel epidemiological evidence that elevated exposure to toxic metals including selenium and lead is associated with higher likelihood of MASLD in American adults. These results highlight the need to reduce toxic metal exposures through regulation of industrial sources and personal hygienic practices. Future research should utilize prospective designs, precisely defined outcomes and diverse biomarkers to elucidate the biological mechanisms linking cumulative metal exposures to MASLD pathogenesis. Our findings underscore the importance of mitigating environmental metal exposures to combat the emerging public health burden of MASLD.

## Data availability statement

The raw data supporting the conclusions of this article will be made available by the authors, without undue reservation.

## Ethics statement

The studies involving humans were approved by the National Center for Health Statistics Research Ethics Review Board, duly approved by the ethical review committee (protocol #2011–17, #2018–01). The patients/participants provided their written informed consent to participate in this study.

## Author contributions

YL: Data curation, Formal analysis, Visualization, Writing – original draft. ZL: Data curation, Methodology, Project administration, Writing – original draft. YC: Data curation, Methodology, Visualization, Writing – original draft. NC: Methodology, Validation, Writing – original draft. RZ: Methodology, Resources, Writing – original draft. XL: Conceptualization, Data curation, Methodology, Supervision, Writing – original draft, Writing – review & editing. WS: Conceptualization, Methodology, Supervision, Writing – review & editing. JL: Conceptualization, Methodology, Supervision, Writing – review & editing.
